# Association of single and multiple-site osteoarthritis with physical and mental health-related quality of life over an 8-year period in US adults: Findings from the osteoarthritis initiative

**DOI:** 10.1097/MD.0000000000042335

**Published:** 2025-05-02

**Authors:** Aqeel M. Alenazi, Mohammed S. Alghamdi, Mohammed M. Alshehri, Bader A. Alqahtani, Maram F. Alanazi, Ragab K. Elnaggar, Ahmed S. Alhowimel, Norah A. Alhwoaimel, Ahmad D. Alanazi, Sattam M. Almutairi, Yasir S. Alshehri, Vishal Vennu, Saud M. Alrawaili, Saad M. Bindawas

**Affiliations:** a Department of Health and Rehabilitation Sciences, Prince Sattam Bin Abdulaziz University, Alkharj, Saudi Arabia; b Department of Medical Rehabilitation Sciences, College of Applied Medical Sciences, Umm Al-Qura University, Makkah, Saudi Arabia; c Physical Therapy Department of Rehabilitation Science, Jazan University, Jazan, Saudi Arabia; d University of Sydney, Sydney, Australia; e Department of Physical Therapy for Pediatrics, Faculty of Physical Therapy, Cairo University, Giza, Egypt; f Department of Rehabilitation Science, Majmaah University, Majmaah, Saudi Arabia; g Department of Physical Therapy, College of Medical Rehabilitation, Qassim University, Buraydah, Saudi Arabia; h Department of Physical Therapy, College of Medical Rehabilitation Sciences, Taibah University, Madinah, Saudi Arabia; i Department of Rehabilitation Sciences, College of Applied Medical Sciences, King Saud University, Riyadh, Saudi Arabia.

**Keywords:** arthritis, cohort studies, health status, mental health, osteoarthritis, polyarthritis, quality of life

## Abstract

This study examined the association of single and multiple-site osteoarthritis (OA) at baseline with physical and mental health-related quality of life (HRQOL) over an 8-year follow-up period in the US adults compared to those at high risk of knee OA. This study is a prospective longitudinal design over 8 years of follow-up. Data from 4796 participants aged between 45 and 79 years were acquired from the osteoarthritis initiative. Based on self-reported physician-diagnosed OA and grade ≥ 2 in either knee using Kellgren and Lawrence grade at baseline, participants were categorized into high risk of knee OA (n = 1560), 1-site OA (n = 1233), 2-site OA (n = 1272), and ≥ 3-site OA (n = 721) groups. Physical and mental components of HRQOL were assessed over an 8-year follow-up period using the 12-item Short Form Health Survey. Two separate generalized estimating equations were used. A total of 4786 participants were included in the final model. Results from the generalized estimating equation showed that participants with 1-site OA (Beta [B] = −1.04, 95% confidence intervals [CI]: (−1.5, −.6), *P* < .001), 2-site OA (B = −2.17, 95% CI: (−2.6, −1.7), *P* < .001), and ≥ 3-site OA (B = −4.98, 95% CI: (−5.7, −4.3), *P* < .001) had significantly declined physical composite score of HRQOL over time than those without OA at baseline after adjustments for covariates, such as age, sex, race, educational status, body mass index, number of comorbidities, physical activity level, and depressive symptoms. Mental composite score of HRQOL had significantly increased across 2-site OA (B = 0.46, 95% CI: (.2,.7), *P* = .001), and ≥ 3-site OA (B = 0.86, 95% CI: (.5, 1.2), *P* < .001) after adjustments for covariates. US adults with single and multiple joint OA at baseline were associated with a decline in physical and an increase in mental HRQOL than those at higher risk of knee OA at baseline.

## 1. Introduction

Osteoarthritis (OA) is a prevalent chronic degenerative disease affecting multiple joints,^[1]^ causing pain, stiffness, limited movement, and impacting health-related quality of life (HRQOL).^[2]^ The prevalence of OA was estimated to be 10% among US adults, and approximately 70% of them have symptoms at other joints suggesting multiple joints OA.^[3–6]^ However, most previous studies have focused on OA or pain with a specific or single joint such as the knee, hip, or hand OA.^[7–9]^ This focus on one site from research and clinical perspectives is due to the high prevalence of single joint OA, and most complaints from patients are on one site during clinical visits. Moreover, a single site of OA could represent different etiology and risk factors from multiple joints of OA.^[10,11]^

Multiple joints with OA can lead to a high clinical burden and poor HRQOL.^[2]^ Previous studies^[12–14]^ have shown reduced HRQOL in individuals with OA, but these studies have limitations as they focus on a single joint, such as the knee, hip, or hand. Other studies^[2,15,16]^ have also shown a link between multiple joints OA, pain, and decreased HRQOL, but these have limitations like cross-sectional design, small sample size, focus on painful joints, and lack of comparison group. However, Hoogeboom et al^[15]^ found joint pain related to comorbidity decreased HRQOL longitudinally but was limited to specific joints similar to previous reports.^[3–6,12–14]^ In addition, recent guidelines have categorized multiple joints OA as a distinct group, offering specific recommendations for nonsurgical interventions.^[17]^

Previous research^[1,2,18]^ utilized a homunculus to identify the number of symptomatic joints. However, this approach to diagnosing OA, focusing on painful sites, may not accurately represent multisite OA, as it may be attributed to multiple locations and generalized form. Patients with multiple joints OA may present with complaints due to the primary symptomatic joint, but they may also have fewer or non-symptomatic other joints. Thus, it is crucial to investigate the relationship between multiple joints of OA and long-term HRQOL, especially among individuals with or at risk of knee OA.

Given the limitations in previous studies,^[3–6,12–15]^ this is the first study to provide a comprehensive approach by combining self-reported and radiographic knee OA to identify multisite joint involvement, including less or non-symptomatic joints. The objective of this study was to examine the association of single and multiple-site OA at baseline with physical and mental HRQOL over an 8-year follow-up period in US adults compared to those at high risk of knee OA at baseline. The study hypothesized that single, particularly multiple joints OA, would be more strongly associated with a decline in physical and mental HRQOL in this population.

## 2. Methods

This study is a secondary analysis of a longitudinal prospective cohort from the osteoarthritis initiative (OAI). A detailed description of the OAI study has been reported at https://nda.nih.gov/oai/. The OAI study involved a total of 4796 participants aged between 45 and 79 at the time of recruitment who were with or at high risk for symptomatic knee OA. individuals who did not have knee OA at baseline but who could potentially develop it during the study period, were classified as being at risk for symptomatic knee OA. Participants in the main study completed 8 visits from baseline to 96 months with variable time intervals. Data collection included biospecimen collection, imaging, and clinical assessments.

In this study and database, all procedures performed involving human participants were in accordance with the ethical standards of the institutional and/or national research committee and with the 1964 Helsinki Declaration and its later amendments or comparable ethical standards. This study was approved by the Institutional Review Board for the University of California, San Francisco and its affiliates (approval number: FWA00000068). The Institutional Review Board approval was also obtained from all 4 clinical sites located at Brown University in Rhode Island, Ohio State University in Columbus, Ohio, University of Maryland/Johns Hopkins University joint center in Baltimore, Maryland, and at the University of Pittsburgh in Pennsylvania. An informed consent was obtained from each participant before the enrollment.

### 2.1. Cohort selection

For the current study, participants were selected from the main study regardless of age, sex, or race. Based on self-reported physician-diagnosed OA at baseline and radiographic knee OA in either knee using Kellgren and Lawrence (KL) grade at baseline (KL ≥ 2), all participants were categorized into a high-risk of knee OA (n = 1560), 1-site OA (n = 1233), 2-site OA (n = 1272), and ≥ 3-site OA (n = 721) groups. Baseline data were used to describe sample demographic and anthropometric characteristics.

### 2.2. Outcome measures

The primary outcome of this study was the HRQOL as measured by the medical outcomes study short-form 12 (SF-12) at baseline and follow-up visits up to 96-month (8 years) visits over 7-time points.^[19]^ The SF-12 is a generic self-report questionnaire of HRQOL and consists of 12 items reflecting an individual’s functioning across 8 health domains. The SF-12 is designed to create composite scores reflecting physical health (Physical Component Summary Scale Score, PCS-12) and mental health (Mental Component Summary Scale Score, MCS-12). Norm-based standardized scores were calculated for the PCS-12 and MCS-12 scales to have a mean of 50 and a standard deviation of 10 in the general US population.^[19]^ A score higher or lower than the norm-based score of 50 indicates better or poorer performance in the PCS-12 and MCS-12, respectively.^[19]^ The SF-12 has established validity and reliability with a wide range of conditions and has been extensively used in research involving individuals with OA.^[19,20]^

### 2.3. Exposure group

Multiple joints OA at baseline visit was the predictor variable. Previous evidence has defined multiple joints OA in multiple approaches, and the majority of the definitions included 3 sites or more of OA.^[1]^ Therefore, after using self-reported physician-diagnosed OA and radiography knee OA (KL ≥ 2) at baseline, 4 categories were established (high risk of knee OA, 1-site OA, 2-site OA, and ≥ 3-site OA) based on previous evidence. Specifically, participants were asked yes/no questions regarding physician-diagnosed OA for common joints in separate questions: “Doctor said you had osteoarthritis/degenerative arthritis in the knee.” The same question was repeated for the hip, hand/finger, back/neck, and a question for some other joints.

### 2.4. Confounders

Confounders for the current study were basic demographics (age, sex, race, education, and body mass index [BMI]) and selected clinical variables (depressive symptoms, physical activity level, number of medications, and number of comorbidities). These confounding variables have been adjusted for in previous similar research.^[21]^ Age was recorded in years. The race variable was categorized into (White vs others such as Black/Asian/Other non-white) because the majority of the sample was of the White race. The level of education was categorized into as high school/less and some college/graduate. BMI was obtained by dividing body mass (kg) by the square of height (m^2^).

Depressive symptoms were assessed by the self-reported 20-item Center for Epidemiologic Studies-Depression Scale.^[22]^ Physical activity was assessed using the Physical Activity in the Elderly Scale (PASE).^[23]^ The number of comorbidities was obtained using an adapted self-reported questionnaire from Charlson Index^[24,25]^ and classified into none, 1, 2, and 3 or more comorbidities.

### 2.5. Data analysis

Descriptive statistics were computed for all baseline demographics and clinical variables. Differences between high risk of knee OA, 1-site OA, 2-site OA, and ≥ 3-site OA were examined using one-way ANOVA and Chi-square using Pearson Chi-Square for testing independence for continuous variables and categorical, respectively. Tukey’s least significant difference was used for post hoc analysis at baseline.

To examine whether OA, particularly multiple sites OA has an impact on physical and mental HRQOL, two separate generalized estimating equations (GEE) modeled with a linear regression analysis were used. A high risk of knee OA, 1-site OA, 2-site OA, and ≥ 3-site OA were the predictor variables. The outcome variables were the physical and mental component summary of HRQOL.

To account for the influence of demographic and clinical variables on the relationship between OA sites and physical and mental HRQOL, two models were created. Model 1 was used to adjust for age, sex, race, and education. Model 2 was used to adjust for variables in model 1 in addition to BMI, depressive symptoms, physical activity, number of medications, and number of comorbidities. A high risk of knee OA was used as the reference.

Assumptions of all statistical analyses were explored to ensure stringent analyses and accurate results. The alpha level was set at 0.05 for all analyses. IBM SPSS for Mac version 25.0 (SPSS Inc. Chicago, IL) was used for all analyses.

## 3. Results

**Figure 1.**
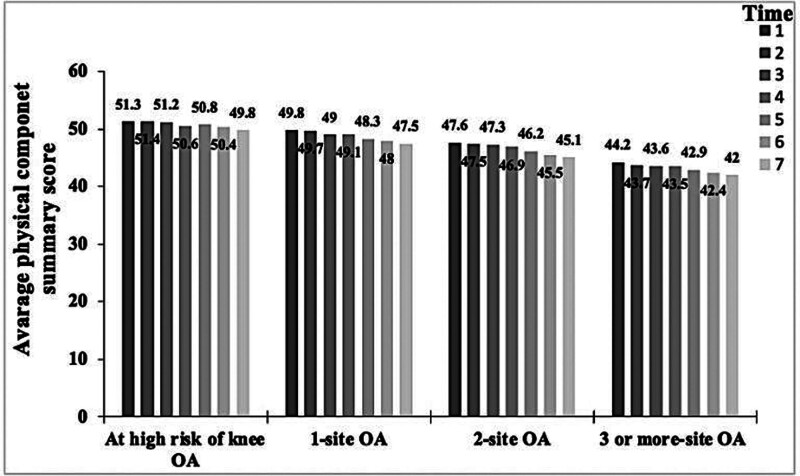
The average physical component summary score for each time point and OA category.

**Figure 2.**
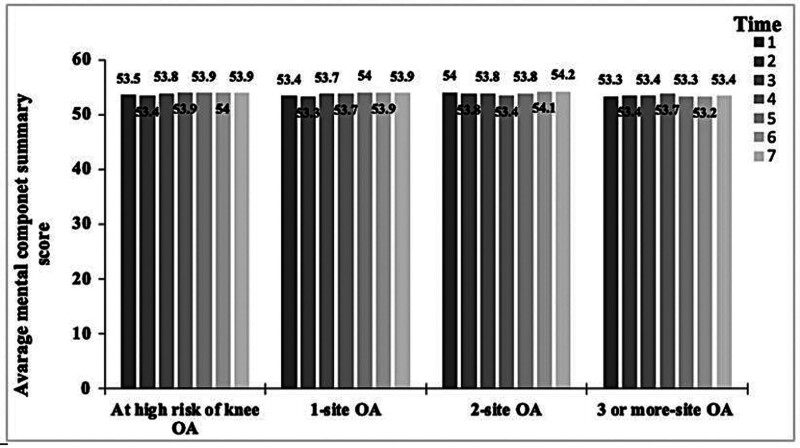
The average mental component summary score for each time point and OA category.

A total of 4786 were included in the final model after full adjustments of the covariates. Baseline characteristics including demographics and clinical variables are shown in Table 1. All participants were categorized into high-risk knee OA, 1-site OA, 2-site OA, and ≥ 3-site OA groups. Approximately 32.5% of the participants did not report the diagnosis of OA at baseline while 15% had at least 3 sites of OA as a diagnosis. All baseline demographics and clinical variables were statistically different between OA groups as shown in Table 1. post hoc analysis using Tukey’s least significant difference showed significant differences between all groups when compared to at-risk in age, BMI, PASE, and PCS. However, MCS was not statistically different between groups when compared to the at-risk group. Center for Epidemiologic Studies-Depression Scale was statistically different between all groups when compared to the at-risk group except for 1-site OA. Means for each OA category for PCS and MCS for HRQOL at baseline and over 7 times of the following-up are shown in Figures 1 and 2, respectively.

**Table 1:** Baseline demographics and clinical characteristics of the study participants in each OA group.

Factors	At high risk of knee OA N = 1560	1 site OA N = 1233	2 sites OA N = 1272	≥ 3 sites OA N = 721	*P* *
Age, years (mean ± SD)	58.49 ± 9	61.27 ± 9	62.69 ± 9	64.02 ± 8	**<.001**
Sex, females, (% within OA)	848 (54.4)	672 (54.5)	771 (60.6)	507 (70.3)	**<.001**
Race, white, (% within OA)	1233 (79)	1032 (83.7)	938 (73.7)	582 (80.7)	**<.001**
BMI, Kg/m^2^ (mean ± SD)	27.47 ± 4.6	28.34 ± 4.4	29.66 ± 5	29.75 ± 4.9	**<.001**
Education, n, (% within OA)					**<.001**
High school/less	218 (14.1)	178 (14.6)	230 (18.2)	144 (20.1)	
Some college/graduate	1324 (85.9)	1043 (85.4)	1036 (81.8)	572 (79.9)	
Depression (mean ± SD)	6.07 ± 6.6	6.39 ± 6.9	6.75 ± 6.8	7.88 ± 7.8	**<.001**
PASE (mean ± SD)	170 ± 83	162 ± 82	156 ± 80	145 ± 79	**<.001**
Comorbidities, (% within OA)					**<.001**
None	1220 (79.6)	913 (75.1)	934 (74.1)	491 (69.0)	
1	198 (12.9)	196 (16.1)	196 (15.6)	131 (18.4)	
2	76 (5.0)	74 (6.1)	93 (7.4)	53 (7.4)	
3 or more	38 (2.5)	32 (2.6)	37 (2.9)	38 (5.3)	
PCS (mean ± SD)	51.3 ± 8	49.7 ± 8.8	47.6 ± 8.9	44. 17 ± 10.2	**<.001**
MCS (mean ± SD)	53.5 ± 7.7	53.4 ± 8	54 ± 7.9	53.3 ± 9.1	.156

Missing cases for the OA category (n = 48).

BMI = body mass index, MCS = mental component summary, OA = osteoarthritis, PASE = Physical Activity Scale for Elderly, PCS = physical component summary.

* *P* indicates the *P*-value that was based on Chi-square for categorical variables or one-way ANOVA for continuous variables.

The results of GEE linear regression for the impact of multiple sites OA on the PCS of HRQOL are shown in Table 2. The results showed that participants with 1-site OA (Beta [B] = −1.04, 95% confidence intervals [CI]: (−1.5, −.6), *P* < .001), 2-site OA (B = −2.17, 95% CI: (−2.6, −1.7), *P* < .001), and ≥ 3-site OA (B = −4.98, 95% CI: (−5.7, −4.3), *P* < .001) had significantly declined PCS of HRQOL over time when compared to those with a high risk of knee OA at baseline after adjustments for age, sex, race, education, BMI, depression, PASE, and number of comorbidities. After correcting for confounders, non-whites, such as blacks, Asians, or other non-whites, significantly had lower PCS scores of 1.96, 95% CI: (−2.5, −1.4), (*P* < .001) than whites. Depression has also been found to be substantially linked with a somewhat lower PCS score of 0.22, 95% CI: (−3, −.19), (*P* < .001). Additionally, PCS of HRQOL values were significantly worse by 2.38, 95% CI: (−2.9, −1.8), 3.04, 95% CI: (−3.9, −2.1), and 4.78, 95% CI: (−6.3, −3.3), points, respectively, for people with 1, 2, and 3 or more comorbidities.

**Table 2:** GEE with linear regression for the association of baseline single and multiple sites OA with physical HRQOL over time.

Factors	Model 1 (n = 4739)			Model 2 (n = 4701)		
	B (95% CI)	SE	*P*	B (95% CI)	SE	*P*
At high risk of knee OA	1.00			1.00		
One site OA	−1.79 (−2.3, −1.3)	0.27	**<.001**	−1.04 (−1.5, −.6)	0.25	**<.001**
Two sites OA	−3.30 (−3.8, −2.7)	0.28	**<.001**	−2.17 (−2.6, −1.7)	0.25	**<.001**
≥three sites OA	−6.80 (−7.5, −6.1)	0.37	**<.001**	−4.98 (−5.7, −4.3)	0.34	**<.001**
Age*	−0.11 (−1, −0.8)	0.01	**<.001**	−0.079 (−1, −.05)	0.01	**<.001**
Sex (males vs females)	−0.16 (−.6,.27)	0.22	.455	0.013 (−.3,.4)	0.20	.95
Race (white vs others**)	−3.72 (−4.3, −3.1)	0.31	**<.001**	−1.96 (−2.5, −1.4)	0.29	**<.001**
Education (high school/less vs some college/ graduate)	2.48 (1.8, 3.2)	0.34	**<.001**	1.19 (.5, 1.8)	0.31	**<.001**
BMI*				−0.38 (−.4, −.3)	0.022	**<.001**
Depression*				−0.22 (−.3, −.19)	0.01	**<.001**
PASE*				0.01 (.011,.015)	0.001	**<.001**
Comorbidities						
None				1.00	1.00	1.00
1				−2.38 (−2.9, −1.8)	0.29	**<.001**
2				−3.04 (−3.9, −2.1)	0.48	**<.001**
3 or more				−4.78 (−6.3, −3.3)	0.78	**<.001**

References were at high risk of knee OA, male sex, white race, high school/less education, and none comorbidities.

95% CI = 95% confidence interval, B = Beta (unstandardized coefficients), BMI = body mass index, OA = osteoarthritis, PASE = Physical Activity for Elderly Scale.

* Others refer to nonwhite, such as Black/Asian/Other nonwhite.

** Indicates that adjustments were made at baseline and over time (7-time points of follow up), and all other factors were at baseline only.

The results of GEE linear regression for the impact of multiple sites OA on the MCS of HRQOL are shown in Table 3. Participants with 1 site of OA (B = 0.16, 95% CI: (−.1,.4), *P* = .229) had no significant increase in the MCS, whilst 2 sites of OA (B = 0.46, 95% CI: (.2,.7), *P* = .001), and 3 sites or more of OA (B = 0.86, 95% CI: (.5, 1.2), *P* < .001) had a significant increase in the MCS of HRQOL over time when compared to those at high risk of knee OA after adjustments for age, sex, race, education, BMI, depression, PASE, and number of comorbidities. Low education levels (high school or less) and depression were significantly associated with poorer mental composite scores of 0.45, 95% CI: (−.8. −.1), (*P* = .003) and 0.83, 95% CI: (−.85, −.81), (*P* < .001).

**Table 3:** GEE with linear regression for the association of baseline single and multiple sites OA with mental HRQOL over time.

Factors	Model 1 (n = 4739)			Model 2 (n = 4701)		
	B (95% CI)	SE	*P*	B (95% CI)	SE	*P*
At high risk of knee OA	1.00			1.00		
One site OA	−0.34 (−.8,.1)	0.24	.158	0.16 (−.1,.4)	0.14	.229
Two sites OA	−0.15 (−6,.3)	0.25	.557	0.46 (.2,.7)	0.14	**.001**
≥three sites OA	−0.70 (−1.3, −.1)	0.31	**.025**	0.86 (.5, 1.2)	0.18	**<.001**
Age*	0.11 (0.09, 0.1)	0.01	**<.001**	0.09 (.07,.09)	0.01	**<.001**
Sex (males vs females)	−0.93 (−1.3, −.6)	0.19	**<.001**	−0.41 (−.6, −.2)	0.11	**<.001**
Race (white vs others**)	−0.77 (−1.3, −.2)	0.28	**.006**	0.48 (.2,.8)	0.16	**.003**
Education (high school/less vs some college/ graduate)	1.63 (1.03, 2.2)	0.31	**<.001**	−0.45 (−.8. −.1)	0.17	**.009**
BMI*				0.07 (.05,.09)	0.01	**<.001**
Depression*				−0.83 (−85, −.81)	0.01	**<.001**
PASE*				0.002 (.001,.003)	0.001	**.004**
Comorbidities						
None				1.00	1.00	1.00
1				0.26 (−06,.5)	0.16	.113
2				0.12 (−4,.6)	0.26	.644
3 or more				0.07 (−.7,.8)	0.39	.85

References were at high risk of knee OA, male sex, white race, high school/less education, and none comorbidities.

95% CI = 95% confidence interval, B = Beta (unstandardized coefficients), BMI = body mass index, OA = osteoarthritis, PASE = Physical Activity for Elderly Scale.

* Indicates that adjustments were made at baseline and over time (7-time points of follow up), and all other factors were at baseline only.

** Others refer to non-white such as Black/Asian/Other non-white.

## 4. Discussion

The current study examined the association of single and multiple-site OA at baseline with physical and mental HRQOL over an 8-year follow-up period in US adults compared to those at high risk of knee OA at baseline. The study hypothesis was partially supported in that participants with self-reported physician-diagnosed single and multiple joints OA at baseline were associated significantly with a decline in the physical but a significant increase in mental HRQOL over 96 months than those with a higher risk of knee OA at baseline after controlling for covariates.

Consistent with the current findings, a recent study found that hand OA at a single site was linked to lower HRQOL in both the general population and hospital patients when compared to individuals without OA.^[26]^ Additionally, the same study found that having both hand and knee OA concurrently further reduced physical HRQOL compared to hand OA alone. Other previous studies supported these findings by concluding that polyarticular OA (i.e., unilateral or bilateral OA in 2 or more joint sites) has a greater impact on physical HRQOL when compared to a single site of hand OA.^[12,27,28]^ In terms of clinical relevance, patients with hand OA alone and patients with both hand and knee OA demonstrated a clinically relevantly lower physical HRQOL than individuals without OA.^[26]^ In the current study, physical HRQOL scores for patients with 2 sites of joint OA as well as those with 3 or more sites of OA were 2.4 and 5.5 points below the norm-based score of 50, respectively.^[19]^ This indicates that these two groups of patients had clinically relevantly lower physical HRQOL. Future studies should seek potential treatment strategies that aim to improve physical HRQOL in patients with OA, especially in those with multiple sites of OA.

The current study indicated that multisite OA was associated with a decline in physical HRQOL that might be attributed to other risk factors such as multisite pain and physical functions. Previous research found that multisite pain was associated with an increased risk of falls^[29,30]^ and declined physical HRQOL.^[31]^ The low physical HRQOL reported in the current study could be partially explained by central sensitization (CS); a condition characterized by an amplified response of the central nervous system to stimuli, resulting in the perception of pain greatly magnified.^[32]^ People with OA who experience severe pain may avoid physical activity that could exacerbate pain. A recent cross-sectional study compared physical function and CS between individuals with unilateral and bilateral knee OA using the Western Ontario and McMaster Universities Arthritis Index and CS Inventory.^[33]^ Their findings showed that the bilateral knee OA group had significantly lower physical function and higher CS compared to the unilateral knee OA group. Furthermore, centrally driven CS might lead to increased pain over time in people with OA. A recent longitudinal study among knee OA patients undergoing total knee arthroplasty (TKA) (n = 223) compared the changes in pain intensity over time. Their results showed that the persistent disturbed somatosensory functioning group had a worse pain score over time especially 1-year post-TKA compared to the normal somatosensory functioning group, suggesting a presence of centrally driven CS in knee OA patients awaiting TKA.^[34]^ These findings highlighted the roles of psychological aspects in CS. A recent study conducted by Aylin Sariyildiz et al^[32]^ examined the relationship between psychosocial parameters, type D personality (combination of negative affectivity and social inhibition), and CS among participants with knee OA revealed that type D personality was significantly associated with higher CS and lower quality of life.

The results of this study revealed a significant increase in the MCS of HRQOL over time in people with two and multiple sites OA compared to those at high risk of knee OA after adjustments for covariates. Thus, while several studies reported that the HRQOL of older individuals with OA is worse in physical and mental aspects than those without OA, the current study found an improvement in mental HRQOL. The possible explanation is that patients with multiple or severe OA might adjust their mental and emotional health expectations and accept the decline in their physical function.^[21]^ The relationship between multiple sites of OA and mental HRQOL requires more investigation to validate the present study’s results related to the increase in mental HRQOL.

The OA usually affects middle-aged and older populations and is often considered to be an inevitable part of aging.^[35]^ The findings of the current study indicate that aging is associated with a greater number of OA joints. With aging, chondrocyte function declines due to cellular senescence, which results in a reduced ability to remodel and maintain articular cartilage.^[36]^ Therefore, these changes may increase joint stiffness and ultimately to an increased susceptibility to the destruction and development of OA.^[36]^

The finding of this study indicates that the prevalence of multiple joint OA increases continuously with being female. A higher percentage of participants who complain of multiple joint OA ≥ 3 is female with 72%. This finding was also reported in different studies^[1,5]^ suggesting that there is a significant association between being female and a higher number of multiple joint OA. There is a possibility that women’s cartilage may be thinner, which could speed up cartilage loss.^[37]^

It was found that non-whites, including blacks, Asians, and other non-whites, had a worse physical HRQOL than whites. Being single, divorced, or widowed was more likely to occur and had a bigger impact on non-whites with lower annual wages,^[38]^ which could be one explanation. Another reason would be that while TKA was less common in this cohort, the negative health effects of the treatment were more common.^[39]^ It’s interesting to note that people with only a high school education or less had worse mental HRQOL, which was in contrast to an earlier study that found that white graduates were more likely to experience mental health issues.^[38]^ However, a prior study found that having depressive symptoms increased the risk of arthritis and that the likelihood of survival dropped at each time point in both males and females, which is comparable to the findings of the present study.^[40]^

The various number of comorbidities have shown a relationship with OA.^[35]^ The underlying mechanism of this relationship is still unclear. However, some potential mechanisms might explain this relationship such as increasing functional disability and obesity during aging.^[41]^ The model for physical HRQOL showed a complex relationship with various forms of OA, unlike the mental HRQOL was influenced by the number of comorbidities for multiple OA in which aging, obesity, and depression have a consistent role for both factors (i.e., physical and mental). This finding was consistent with a previous study that found an increasing number of morbidities associated with more limitations in activities for people with OA.^[42]^ In addition, the findings support previous evidence that explained depression as a psychological factor for low physical function^[42]^ and considered one of the highest risks for OA.^[43]^ Previous investigations related to common comorbidities associated with OA found diabetes as a risk for declined gait speed, and gait speed predicted diabetes incidence in people with arthritis.^[44–48]^ Current knowledge on OA comorbidities focuses on the distribution and impact of health in future work is needed to establish extensive research designs for confirmed mechanisms, longitudinal effectiveness, and preventive strategies.

The current study has several strengths, including the design of the present study it’s a longitudinal study with a large sample size, which helps to understand the impact of multiple joints OA on HRQOL, notably among people without radiographic knee OA at baseline. One of the main limitations of the study is that due to the nature of the study design, controlling for other covariates was not feasible. Therefore, the lack of control for unmeasured covariates remains a plausible explanation for some of the current findings. Another limitation was the diagnosis of OA in the study sample was based on a reliable and valid self-reported method in addition to KL grades for knee OA. However, using an objective measure, such as a radiographic diagnosis of OA for all joints as it’s considered the gold standard, will provide more reliable results. This study was limited to the multisite involvement of OA rather than joint locations to understand the generalized impact of multisite OA on HRQOL. Therefore, future research should examine the association of specific locations on HRQOL in this population. In addition, HRQOL was not evaluated using SF-36 or other OA-specific measures because of unavailability. Therefore, results should be interpreted with caution because SF-12 measures general quality of life rather than OA-related quality of life even when comorbidities and other variables that could affect general quality of life have been taken into account in analyses. Future studies are warranted to investigate the clusters of objectively diagnosed OA sites and their relationship with HRQOL by addressing all these limitations.

## 5. Conclusion

This study found that US adults with OA, particularly multiple joints OA were more strongly associated with a decline in physical and an increase in mental HRQOL over time compared to those with a higher risk of knee OA at baseline. Future research should explore potential treatment strategies that aim to improve physical HRQOL in this population, especially in those with multiple joints OA.

## Acknowledgments

The authors extend their appreciation to Prince Sattam bin Abdulaziz University for funding this research work through the project number (PSAU/2025/R/1446). The OAI is a public-private partnership comprised of 5 contracts (N01-AR-2-2258; N01-AR-2-2259; N01-AR-2-2260; N01-AR-2-2261; and N01-AR-2-2262) funded by the National Institutes of Health, a branch of the Department of Health and Human Services, and conducted by the OAI Study Investigators. Private funding partners include Merck Research Laboratories; Novartis Pharmaceuticals Corporation, GlaxoSmithKline; and Pfizer, Inc. Private sector funding for the OAI is managed by the Foundation for the National Institutes of Health. This manuscript was prepared using an OAI public use dataset and does not necessarily reflect the opinions or views of the OAI investigators, the NIH, or the private funding partners.

## Author contributions

**Conceptualization:** Mohammed S. Alghamdi, Mohammed M. Alshehri, Bader A. Alqahtani, Maram F. Alanazi, Ragab K. Elnaggar, Ahmed S. Alhowimel, Norah A. Alhwoaimel, Ahmad D. Alanazi, Sattam M. Almutairi, Yasir S. Alshehri, Saud M. Alrawaili, Saad M. Bindawas.

**Data curation:** Aqeel M. Alenazi, Ragab K. Elnaggar, Norah A. Alhwoaimel, Vishal Vennu.

**Formal analysis:** Aqeel M. Alenazi, Bader A. Alqahtani, Ahmad D. Alanazi, Yasir S. Alshehri.

**Investigation:** Mohammed S. Alghamdi, Ahmed S. Alhowimel, Sattam M. Almutairi, Yasir S. Alshehri, Vishal Vennu, Saud M. Alrawaili, Saad M. Bindawas.

**Methodology:** Mohammed M. Alshehri, Bader A. Alqahtani, Maram F. Alanazi, Ragab K. Elnaggar, Ahmed S. Alhowimel, Norah A. Alhwoaimel, Ahmad D. Alanazi, Sattam M. Almutairi, Yasir S. Alshehri, Vishal Vennu, Saud M. Alrawaili, Saad M. Bindawas.

**Resources:** Bader A. Alqahtani, Saud M. Alrawaili.

**Supervision:** Aqeel M. Alenazi, Ragab K. Elnaggar, Vishal Vennu, Saad M. Bindawas.

**Writing – original draft:** Aqeel M. Alenazi, Mohammed S. Alghamdi, Mohammed M. Alshehri, Bader A. Alqahtani, Maram F. Alanazi, Ragab K. Elnaggar, Ahmed S. Alhowimel, Norah A. Alhwoaimel, Ahmad D. Alanazi, Sattam M. Almutairi, Yasir S. Alshehri, Vishal Vennu, Saad M. Bindawas.

**Writing – review & editing:** Mohammed S. Alghamdi, Mohammed M. Alshehri, Bader A. Alqahtani, Maram F. Alanazi, Ragab K. Elnaggar, Ahmed S. Alhowimel, Norah A. Alhwoaimel, Ahmad D. Alanazi, Sattam M. Almutairi, Yasir S. Alshehri, Vishal Vennu, Saud M. Alrawaili, Saad M. Bindawas.

## References

[1] Gullo TR Golightly YM Cleveland RJ., eds. Defining multiple joint osteoarthritis, its frequency and impact in a community-based cohort. Seminars in Arthritis and Rheumatism. Elsevier; 2019. 10.1016/j.semarthrit.2018.10.001 PMC6456431 30390991

[2] Cuperus N Vlieland TPV Mahler EA Kersten CC Hoogeboom TJ van den Ende CH. The clinical burden of generalized osteoarthritis represented by self-reported health-related quality of life and activity limitations: a cross-sectional study. Rheumatol Int. 2015;35:871–7. 25300731 10.1007/s00296-014-3149-1

[3] Keenan A Tennant A Fear J Emery P Conaghan PG. Impact of multiple joint problems on daily living tasks in people in the community over age fifty‐five. Arthritis Care Res. 2006;55:757–64. 10.1002/art.22239 17013823

[4] Croft P Jordan K Jinks C. “Pain elsewhere” and the impact of knee pain in older people. Arthritis Rheumatism. 2005;52:2350–4. 16052574 10.1002/art.21218

[5] Badley EM Wilfong JM Yip C Millstone DB Perruccio AV. The contribution of age and obesity to the number of painful joint sites in individuals reporting osteoarthritis: a population-based study. Rheumatology (Oxford, England). 2020;59:3350–7. 32306046 10.1093/rheumatology/keaa138 PMC7590415

[6] Felson DT Niu J Quinn EK. Multiple nonspecific sites of joint pain outside the knees develop in persons with knee pain . Arthritis Rheumatol. 2017;69:335–42. 27589036 10.1002/art.39848 PMC5292971

[7] Vina ER Kwoh CK. Epidemiology of osteoarthritis: literature update. Curr Opin Rheumatol. 2018;30:160–7. 29227353 10.1097/BOR.0000000000000479 PMC5832048

[8] Bijlsma JW Berenbaum F Lafeber FP. Osteoarthritis: an update with relevance for clinical practice. Lancet (London). 2011;377:2115–26. 10.1016/S0140-6736(11)60243-2 21684382

[9] Neogi T Zhang Y. Epidemiology of osteoarthritis. Rheum Dis Clin North Am. 2013;39:1–19. 23312408 10.1016/j.rdc.2012.10.004 PMC3545412

[10] Van Spil W Welsing P Bierma-Zeinstra S. The ability of systemic biochemical markers to reflect presence, incidence, and progression of early-stage radiographic knee and hip osteoarthritis: data from CHECK. Osteoarthritis Cartilage. 2015;23:1388–97. 25819579 10.1016/j.joca.2015.03.023

[11] Alenazi AM Alothman S Alshehri MM. The prevalence of type 2 diabetes and associated risk factors with generalized osteoarthritis: a retrospective study using ICD codes for clinical data repository system. Clin Rheumatol. 2019;38:3539–47. 31392561 10.1007/s10067-019-04712-0

[12] Slatkowsky‐Christensen B Mowinckel P Loge JH Kvien TK. Health‐related quality of life in women with symptomatic hand osteoarthritis: a comparison with rheumatoid arthritis patients, healthy controls, and normative data. Arthritis Care Res. 2007;57:1404–9. 10.1002/art.23079 18050180

[13] Pereira D Severo M Santos RA. Knee and hip radiographic osteoarthritis features: differences on pain, function and quality of life. Clin Rheumatol. 2016;35:1555–64. 26445941 10.1007/s10067-015-3087-7

[14] Michon M Maheu E Berenbaum F. Assessing health-related quality of life in hand osteoarthritis: a literature review. Ann Rheum Dis. 2011;70:921–8. 21398333 10.1136/ard.2010.131151

[15] Hoogeboom TJ Den Broeder AA De Bie RA Van Den Ende CH. Longitudinal impact of joint pain comorbidity on quality of life and activity levels in knee osteoarthritis: data from the osteoarthritis initiative. Rheumatology (Oxford). 2013;52:543–6. 23204552 10.1093/rheumatology/kes314 PMC3716330

[16] Finney A Dziedzic KS Lewis M Healey E. Multisite peripheral joint pain: a cross-sectional study of prevalence and impact on general health, quality of life, pain intensity and consultation behaviour. BMC Musculoskelet Disord. 2017;18:1–8. 29246141 10.1186/s12891-017-1896-3 PMC5732469

[17] Bannuru RR Osani M Vaysbrot E. OARSI guidelines for the non-surgical management of knee, hip, and polyarticular osteoarthritis. Osteoarthritis Cartilage. 2019;27:1578–89. 31278997 10.1016/j.joca.2019.06.011

[18] Cimmino MA Scarpa R Caporali R Parazzini F Zaninelli A Sarzi-Puttini P. Body mass and osteoarthritic pain: results from a study in general practice. Clin Exp Rheumatol. 2013;31:843–9. 24144227

[19] Ware JE Jr Kosinski M Keller SD. A 12-item short-form health survey: construction of scales and preliminary tests of reliability and validity. Med Care. 1996;34:220–33. 8628042 10.1097/00005650-199603000-00003

[20] Jenkinson C Layte R Jenkinson D. A shorter form health survey: can the SF-12 replicate results from the SF-36 in longitudinal studies? J Public Health Med. 1997;19:179–86. 9243433 10.1093/oxfordjournals.pubmed.a024606

[21] Wilson R Blakely T Abbott JH. Radiographic knee osteoarthritis impacts multiple dimensions of health-related quality of life: data from the osteoarthritis initiative. Rheumatology (Oxford). 2018;57:891–9. 29481663 10.1093/rheumatology/key008 PMC6251551

[22] Radloff LS. The CES-D scale: a self-report depression scale for research in the general population. Appl Psychol Meas. 1977;1:385–401.

[23] Washburn RA Smith KW Jette AM Janney CA. The physical activity scale for the elderly (PASE): development and evaluation. J Clin Epidemiol. 1993;46:153–62. 8437031 10.1016/0895-4356(93)90053-4

[24] Katz JN Chang LC Sangha O Fossel AH Bates DW. Can comorbidity be measured by questionnaire rather than medical record review? Med Care. 1996;34:73–84. 8551813 10.1097/00005650-199601000-00006

[25] Charlson ME Pompei P Ales KL MacKenzie CR. A new method of classifying prognostic comorbidity in longitudinal studies: development and validation. J Chronic Dis. 1987;40:373–83. 3558716 10.1016/0021-9681(87)90171-8

[26] Loef M Damman W de Mutsert R Rosendaal FR Kloppenburg M. Health-related quality of life in patients with hand osteoarthritis from the general population and the outpatient clinic. J Rheumatol. 2020;47:1409–15. 31787601 10.3899/jrheum.190781

[27] Lombnæs GO Magnusson K Østerås N Nordsletten L Risberg MA Hagen KB. Distribution of osteoarthritis in a Norwegian population-based cohort: associations to risk factor profiles and health-related quality of life. Rheumatol Int. 2017;37:1541–50. 28451795 10.1007/s00296-017-3721-6

[28] Moe RH Grotle M Kjeken I Hagen KB Kvien TK Uhlig T. Disease impact of hand OA compared with hip, knee and generalized disease in specialist rheumatology health care. Rheumatology. 2012;52:189–96. 22923755 10.1093/rheumatology/kes215

[29] Alenazi AM. Multisite pain is longitudinally associated with an increased risk of fall among older adults with or at risk of knee osteoarthritis: data from the osteoarthritis initiative [published online ahead of print May 1, 2025]. Am J Phys Med Rehabil. doi:10.1097/PHM.0000000000002650. 10.1097/PHM.0000000000002650 39642296

[30] Alrawaili SM Alkhathami KM Elsehrawy MG Obaidat SM Alhwoaimel NA Alenazi AM. Multisite pain and intensity were associated with history fall among older adults: a cross-sectional study. J Multidiscip Healthc. 2024;17:1241–50. 38524864 10.2147/JMDH.S449531 PMC10960544

[31] Alqahtani BA Alenazi AM. Multisite musculoskeletal pain is associated with long-term declined physical quality of life and knee-related quality of life in older adults with or at risk of knee osteoarthritis. Medicina (Kaunas). 2024;60:1305. 39202586 10.3390/medicina60081305 PMC11356253

[32] Sariyildiz A Coskun Benlidayi I Olmez Engizek S Deniz V. The relation of psychological status and type D personality with central sensitization in knee osteoarthritis: everything is in your mind! Rheumatol Int. 2023;43:2261–9. 37776500 10.1007/s00296-023-05471-7

[33] Oliveira LAS Pontes-Silva A Damasceno KLB. Comparison between pain intensity, functionality, central sensitization, and self-efficacy in individuals with unilateral or bilateral knee osteoarthritis: a cross-sectional study. Rev Assoc Med Bras (1992). 2022;68:1048–52. 36134833 10.1590/1806-9282.20220170 PMC9574988

[34] Vervullens S Meert L Smeets R Verbrugghe J Verdonk P Meeus M. Does pain intensity after total knee arthroplasty depend on somatosensory functioning in knee osteoarthritis patients? A prospective cohort study. Clin Rheumatol. 2024;43:2047–59. 38668988 10.1007/s10067-024-06976-7 PMC11111543

[35] Pickering M-E Chapurlat R. Where two common conditions of aging meet: osteoarthritis and sarcopenia. Calcif Tissue Int. 2020;107:203–11. 32424600 10.1007/s00223-020-00703-5

[36] Jørgensen AEM Kjær M Heinemeier KM. The effect of aging and mechanical loading on the metabolism of articular cartilage. J Rheumatol. 2017;44:410–7. 28250141 10.3899/jrheum.160226

[37] Hudelmaier M Glaser C Hohe J. Age‐related changes in the morphology and deformational behavior of knee joint cartilage. Arthritis Rheumatism. 2001;44:2556–61. 11710712 10.1002/1529-0131(200111)44:11<2556::aid-art436>3.0.co;2-u

[38] Vennu V Abdulrahman TA Alenazi AM Bindawas SM. Annual income, age, marital status, and smoking influence healthcare access among American minorities and Caucasians with knee osteoarthritis. Int J Healthcare Manage. 2023;17:186–94.

[39] Zhang W Lyman S Boutin-Foster C. Racial and ethnic disparities in utilization rate, hospital volume, and perioperative outcomes after total knee arthroplasty. J Bone Joint Surg Am. 2016;98:1243–52. 27489314 10.2106/JBJS.15.01009

[40] Vennu V Misra H Misra A. Depressive symptoms and the risk of arthritis: a survival analysis using data from the osteoarthritis initiative. Indian J Psychiatry. 2019;61:444–50. 31579152 10.4103/psychiatry.IndianJPsychiatry_241_18 PMC6767814

[41] Suri P Morgenroth DC Hunter DJ. Epidemiology of osteoarthritis and associated comorbidities. PM R. 2012;4:S10–9. 22632687 10.1016/j.pmrj.2012.01.007

[42] van Dijk GM Veenhof C Schellevis F. Comorbidity, limitations in activities and pain in patients with osteoarthritis of the hip or knee. BMC Musculoskelet Disord. 2008;9:1–10. 18582362 10.1186/1471-2474-9-95 PMC2453124

[43] Swain S Sarmanova A Coupland C Doherty M Zhang W. Comorbidities in osteoarthritis: a systematic review and meta‐analysis of observational studies. Arthritis Care Res. 2020;72:991–1000. 10.1002/acr.24008 31207113

[44] Alenazi AM Alqahtani B Alshehri MM. 1462-P: baseline gait speed can predict diabetes incidence in individuals with or at risk of knee osteoarthritis: a longitudinal study using data from the osteoarthritis initiative. Am Diabetes Assoc. 2020;69:1462.

[45] Alenazi AM Alqahtani BA Vennu V. Gait speed as a predictor for diabetes incidence in people with or at risk of knee osteoarthritis: a longitudinal analysis from the osteoarthritis initiative. Int J Environ Res Public Health. 2021;18:4414. 33919455 10.3390/ijerph18094414 PMC8122394

[46] Alenazi AM Alshehri MM Alothman S. The association of diabetes with knee pain locations, pain while walking, and walking speed: data from the osteoarthritis initiative. Phys Ther. 2020;100:1977–86. 32750122 10.1093/ptj/pzaa144 PMC7596886

[47] Alenazi AM Alshehri MM Alothman S. Diabetes is associated with slow walking speed in people with knee osteoarthritis: 91: board #7 May 29 9:30 AM - 11:30 AM. Med Sci Sports Exercise. 2019;51:13–13.

[48] Alenazi AM Alshehri MM Alqahtani BA Alanazi AD Bindawas SM. Combined diabetes and arthritis are associated with declined gait speed. Clin Rheumatol. 2020;40:1593–8. 32856200 10.1007/s10067-020-05370-3

